# Repeated evolution of photoperiodic plasticity by different genetic architectures during recurrent colonizations in a butterfly

**DOI:** 10.1098/rspb.2024.2195

**Published:** 2025-02-12

**Authors:** Anna B. Shoshan, Ugo Pindeler, Christopher W. Wheat, Karl Gotthard

**Affiliations:** ^1^ Department of Zoology, Stockholm University, Stockholm, Sweden; ^2^ Bolin Centre for Climate Research, Stockholm University, Stockholm, Sweden

**Keywords:** diapause, genotype-phenotype map, parallel evolution, Z-linkage, plasticity, colonization

## Abstract

In cases of recurrent colonizations of similar habitats from the same base population, it is commonly expected that repeated phenotypic adaptation is caused by parallel changes in genetic variation. However, it is becoming increasingly clear that similar phenotypic variation may also evolve by alternative genetic pathways. Here, we explore the repeated evolution of photoperiodic plasticity for diapause induction across Swedish populations of the speckled wood butterfly, *Pararge aegeria*. This species has colonized Scandinavia at least twice, and population genomic results show that one of the candidate regions associated with spatial variation in photoperiodism is situated on the Z-chromosome. Here, we assay hybrid crosses between several populations that differ in photoperiodic plasticity for sex-linked inheritance of the photoperiodic reaction norm. We find that while a cross between more distantly related populations from the two different colonization events shows strong sex-dependent inheritance of photoperiodic plasticity, a cross between two more closely related populations within the oldest colonization range shows no such effect. We conclude that the genotype–phenotype map for photoperiodic plasticity varies across these populations and that similar local phenotypic adaptation has evolved during recurrent colonization events by partly non-parallel genetic changes.

## 1. Introduction

Environmental conditions and resource availability often vary considerably across a species range resulting in spatial variation in natural selection and local adaptation [1–3]. Parallel changes in environmental conditions, for instance, due to repeated dispersal events, are expected to lead to repeated evolution of similar adaptive phenotypic differences between populations [4,5]. A common expectation is that such repeated phenotypic evolution is due to parallel changes in genetic variation (i.e. ‘gene reuse’ [6,7]). However, it is possible that more complex phenotypic adaptations may evolve in parallel by different, alternative changes in genetic variation [8]. If so, the genotype–phenotype map will vary across populations.

The evolution of similar phenotypes across similar environments is a typical signature of natural selection [5,6,9,10] and is common across all life [11–17]. A classic example is the repeated evolution of a reduction in armour plates across the body when the marine three-spined stickleback (*Gasterosteus aculeatus*) recurrently has colonized freshwater lakes and streams [18]. For this trait, the decrease in armour plates was found to be predominantly caused by repeated fixation of the same alleles in one single gene [19]. However, similar cases of repeated phenotypic evolution are instead partly due to non-parallel genetic changes. For instance, the oldfield mouse (*Peromyscus polionotus*) in the southeastern United States has two coat colour morphs, a dark morph for mainland camouflage and a light morph for sandy coastal regions. Populations on Florida’s Gulf Coast and Atlantic coasts have independently adapted to sandy environments, and although they share similar light-coloured coats, the underlying genetic mechanisms are partly different [20,21].

Range expansion across latitudes is a common process that is expected to happen in parallel across regions and has led to the evolution of similar adaptive phenotypic differences, both within and between species [22–24]. An example is plasticity for diapause induction in insects. Diapause is a type of dormancy that allows insects to survive periods unsuitable for growth and reproduction, such as cold winters [25]. Winter diapause has evolved repeatedly as insects have expanded and shifted their range from the southern tropical regions into the northern temperate zone [26,27], and the life stage(s) where diapause can be expressed also vary across species [28]. The plastic induction of winter diapause is strongly associated with the ability of many temperate insects to produce several generations per year [29]. The expression of either non-diapause or diapause developmental pathways is controlled by adaptive plasticity in response to seasonal cues such as photoperiod and temperature, and the seasonal timing of this decision will determine the number of annual generations of a given population [25,30]. The decision to induce winter diapause is typically made in late summer or early autumn, well before the onset of cold conditions [31,32]. Since the length of the growth season varies depending on latitude and altitude, populations of the same species often differ in the photoperiod that induces diapause, and as a consequence also in the number of annual generations [32–36]. A frequently used metric to assess this variation across populations is the critical photoperiod, which is the daylength where 50% of a population enters diapause [37–39].

While the diapause phenotype appears conserved across insect species, the genetic background of plasticity for diapause induction seems to vary [40]. For instance, a study of the flesh fly (*Sarcophaga bullata*) suggests that the diapause response is controlled by a single gene or a small gene cluster [41] while a study of the speckled wood butterfly (*Pararge aegeria*) indicates a combination of several genes with high and low effect size [42]. Additionally, some studies find that the genetic background is sex-linked [42–44] while others find it to be almost exclusively determined by autosomal alleles [41,45,46]. However, whether the genetic architecture of diapause induction may also differ among populations of the same species is not well known [8].

Interestingly, variation in the seasonal timing of diapause is often associated with variation at circadian clock genes, which often are found both on sex chromosomes and on autosomes [41,42,47–49]. The circadian clock is a mechanism to time the day–night cycle and although there is substantial variation across species, the timer is often also associated with photoperiodism [50–55]. It has been proposed that the circadian clock’s light–dark cycle may provide information about the change in daylength across time necessary for photoperiodism [56,57]. Still, the potential connection between the circadian clock and photoperiodism of diapause induction is not well understood [58,59] and the clock genes that are associated with the diapause response vary between species [41,42,49]. If the circadian clock can contribute in different ways to photoperiodism between species, then it may also contribute differently to variation among populations.

**Figure 1.**
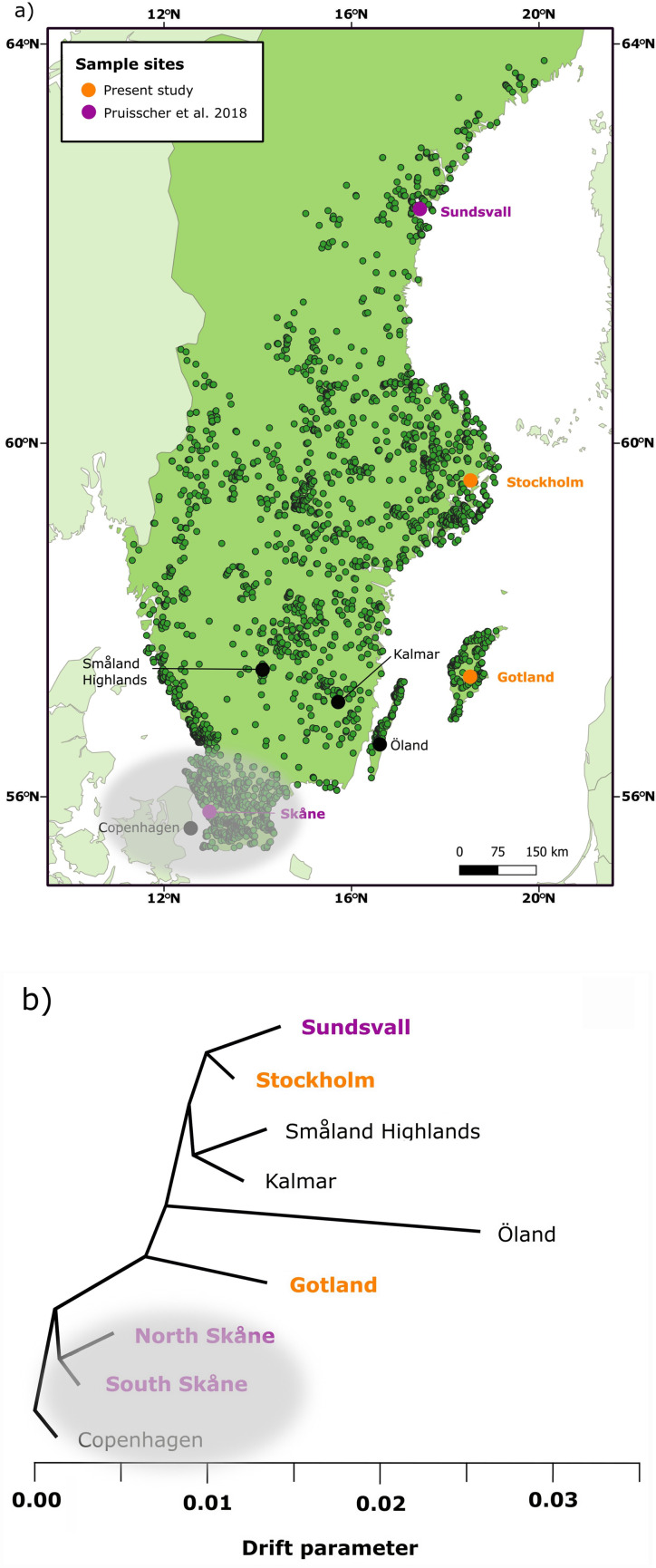
(a) Map of Sweden highlighting the sample sites. The green points are where adult *P. aegeria* have been observed from January 2017 to May 2023 according to citizen science data (Artportalen.se). The orange points are the sample sites that have been used for population crosses in the present study (Stockholm and Gotland) while the purple points are the sample sites from Pruisscher *et al*.'s 2018 study [42] (Sundsvall and Skåne). The grey bubble covering southern Sweden and parts of Denmark indicates populations from the most recent colonization event. (b) A Treemix plot showing the evolutionary relationship of seven populations of *P. aegeria* in Sweden based on population genomic data where branch length indicates amount of genetic drift (based on figure 1*b* in [62]).

In this study, we aim to investigate if variation in induction of winter diapause has the same genetic background across two separate colonizations of Sweden by the speckled wood butterfly (*P. aegeria*). The genetic relationships between populations and entomological records both suggest that the species has expanded into Sweden twice since the last glaciation. Present-day central and northern Swedish populations are descendants from an early expansion that occurred before there were any written records, while the most southern populations are the result of a second, more recent expansion less than 100 years ago (approx. year 1930) [60,61] (figure 1). By comparing the underlying genetic variation causing a difference in the response to photoperiod between and within these two present-day distributions, it is possible to assess whether repeated adaptive evolution of plasticity for diapause induction relies on the same or different genetic variation. Importantly, *P. aegeria* has a well-studied cline in the critical photoperiods in Sweden [35] that is regulated by several genes [42].

A previous study of the genetic background to adaptive divergence in the critical photoperiod investigated population crosses between two distantly related populations of *P. aegeria* in Sweden and found a strong association between phenotypic variation in the critical photoperiod and two genomic regions, one being sex-linked (on the Z-chromosome) and the other autosomal, that included the central clock genes period and timeless, respectively [42]. These two populations originated from the two different expansion events into Sweden, one from the oldest colonization (a population at the species most northern range margin, Sundsvall region) and one from the more recent colonization (the most southern population, Skåne region) [42]. Here, we test if a similar phenotypic divergence in diapause induction between two different populations, both originating from the oldest expansion event, shows a similar sex-linked inheritance. We did this by exploring phenotypic differences of the reciprocal F1 crosses between these two more closely related populations and compared the results to the original data of the study by Pruisscher *et al.* [42]. A lack of sex-linkage of the diapause response between these two closely related populations from the initial colonization would suggest that variation in critical photoperiod within the oldest expansion is not dependent on variation at the Z-chromosome and therefore does not rely entirely on the same genetic variation for diapause induction as the second colonization into Sweden.

## 2. Methods

The speckled wood butterfly (*P. aegeria*) is distributed across Europe and North Africa [63]. It is a woodland species whose larvae feed on a diversity of grass species, including *Dactylis glomerata* and *Poa annua* [64]. The species is a well-studied model organism in regards to seasonal adaptation and voltinism [35,42,64–67]. In Sweden, it is distributed across a latitudinal cline with genetically distinct populations showing local adaptation in voltinism [29,65]. The variation in voltinism is controlled by seasonal plasticity in response to photoperiod and, in particular, to the induction of diapause in response to shorter days in late summer [29,68]. The populations across Sweden have different critical photoperiods [35], which is defined as the photoperiod resulting in 50% of the individuals in a population entering diapause [51]. From the southern part of the Swedish distribution (Skåne) to the northern range margin (Sundsvall), the critical photoperiod differs by approximately 2.7 h (16.3–19 h) [35]. The variation in critical photoperiod is accompanied by variation in voltinism, where most of the southern populations, as well as the two island populations (Öland and Gotland) have two annual generations (bivoltine), while all other populations only have one generation per year (univoltine) [29]. While *P. aegeria* is capable of winter diapause in both the larval and pupal stages [69], it mainly diapauses as a pupa in Sweden [66].

**Figure 2.**
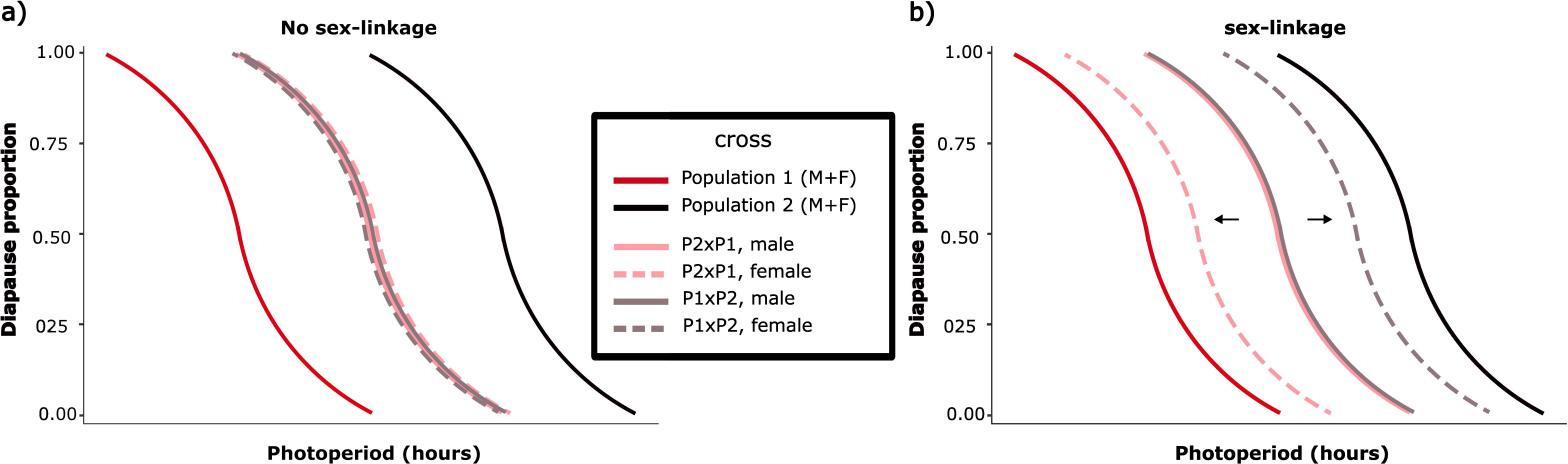
Illustration of expected results when analysing diapause proportions of reciprocal crosses with (a) no sex-linkage or (b) sex-linkage explaining part of the difference in the diapause response between the two original populations. The F1 male reciprocal crosses are expected to have a reaction norm that is intermediate to the two original populations. The same is expected of the reciprocal female crosses if there is no sex-linkage. If the reciprocal female crosses differ, it would indicate that genetic variation at the Z-chromosome influences the difference in the critical photoperiod of the two original populations.

The two populations of *P. aegeria* that were the main focus of this study are from just north of Stockholm and from the island of Gotland, respectively. They are situated approximately 245 km apart and are relatively closely related (figure 1), and population genomic analysis suggests that there has also been more recent gene flow from Stockholm to Gotland [62]. Despite this, the two populations have a substantial difference in their critical photoperiod of around 1.4 h (Gotland ≈ 17.2 h and Stockholm ≈ 18.6 h) [35]. The main aim of the present study was to investigate if this difference in the critical photoperiod is dependent on genetic variation on the Z-chromosome, which was found to be the case in the comparison of the more distantly related populations from southern Sweden and the northern range margin in Sweden. For this, we created F1 reciprocal hybrid crosses of the Stockholm and Gotland populations. As females are the heterogametic sex in butterflies (ZW), the females of the F1 crosses only have one Z-chromosome that they have inherited from their father, while they are heterozygous for all autosomal chromosomes. The males are the homogametic sex (ZZ) and are consequently heterozygous for all chromosomes in these F1 hybrids. Therefore, if genetic variation on the Z-chromosome to some degree influences the differences between our two original populations, we expect that the diapause response of the F1 females should differ between the two reciprocal hybrid crosses (i.e. father from Stockholm or Gotland; figure 2).

In June 2021, wild mated females of the speckled wood butterfly (*P. aegeria*) were collected from the Baltic Island of Gotland (57.40° N, 18.52° E; 15 wild females) and from the mainland north of Stockholm (Riala) (59.60° N, 18.54° E; 16 wild females). All females were allowed to oviposit individually in 0.5 l plastic cups with access to the host plant, *P. annua*. At hatching, larvae were reared in 30 × 40 × 50 cm net cages in long-day conditions, causing non-diapause development in both populations (22 : 2 h light : dark, 23°C). Within each cage were up to 15 larvae from each of two to three females from the same population, and they were fed ad libitum a second host plant, *D. glomerata*. The resulting adults (157 Gotland (G) and 99 Stockholm (S)) were used to produce F1 offspring of the two natural populations (SS and GG) as well as reciprocal F1 population hybrids (SG and GS, female first). In total, 13 GG, 15 SS, 22 GS and 21 SG unique crosses were successfully completed. The F1 larvae from all crosses were placed individually in 0.5 l plastic cups on the grass *P. annua* and distributed among five climate cabinets with a constant temperature of 18°C and one of the following photoperiods: 17 h, 17.4 h, 17.8 h, 18.2 h and 18.6 h, which are covering the range of photoperiods intermediate to the critical photoperiods of the two original populations (Gotland ≈ 17.2 h and Stockholm ≈ 18.6 h) [35]. All climate cabinets contained a datalogger (HOBO Pendant MX2202), confirming the programmed photoperiod and low variation in temperature throughout the experiment. Each photoperiod treatment contained 15 GG (from 13 families), 16 SS (15 families), 44 GS (21 families) and 46 SG (21 families) individuals evenly distributed from the individual crosses and with a maximum of three siblings per treatment (electronic supplementary material, tables S1 and S2). All individuals were checked daily. Whenever the plant had deteriorated in quality or had been consumed by a larva, the larva was supplied with fresh host plants, ensuring ad libitum food throughout the experiment. For each individual, the following was recorded: date of larval hatching, date of pupation, pupal weight, sex, adult eclosion date and adult weight. Pupal and adult weight were measured on a Precisa 205 A SCS balance with a precision of 0.1 mg. Pupal weight was recorded 2 days after pupation to secure sufficient hardening of the cuticle before handling. Sex of each individual was determined in the pupal stage and corroborated in all adults that eclosed [70].

The populations of *P. aegeria* in this study enter winter diapause in the pupal stage [35]. However, the decision to enter diapause is a two-step process and involves two major deviations from non-diapause development. First, a decision to prolong larval development in order to time pupation to the start of winter, followed by a second and final decision to enter pupal diapause and postpone adult eclosion until the following spring [29,65,68]. These are two separate plastic developmental decisions that each have their own photoperiodic reaction norms [29,65] and can evolve independently [71]. Under natural conditions, individuals that decide to prolong larval development time will likely always enter diapause as pupae. This is because such prolonged development typically causes last-instar larvae to make the final pupal diapause decision during the predictably shorter photoperiods of late summer and autumn. Consequently, the reaction norm for the decision to prolong larval development time is shifted to slightly longer photoperiods than the decision to enter pupal diapause [68,71]. However, the two decisions are largely physiologically independent. Previous experiments have demonstrated that the pupal diapause decision can be expressed independently of the larval developmental decision when individuals are exposed to a rapidly increased or decreased photoperiod, conditions that would not normally occur in the wild [65]. Therefore, we here analysed the two decisions separately. The decision of larval development time (hereafter termed ‘larval decision’) is made throughout larval development and is especially expressed in the third and fourth larval instars [29,65]. Larvae that enter non-diapause development typically develop quickly through the larval stage and pupate within 20−37 days after egg hatch (at 17°C−18°C) [29,42] whereas individuals that enter the diapause pathway have markedly longer larval development [29,42] (electronic supplementary material, figure S1). Therefore, in line with previous studies, the larval decision was scored as non-diapause development when larval development time was shorter than 40 days. The decision to enter pupal diapause is finalized in the fourth and last larval instar, just before pupation [65,70]. Individuals that enter non-diapause pupal development will spend 12−20 days in the pupal stage (at 18°C) while pupal diapause lasts up to eight months [72]. Also, in this case, we followed earlier studies and scored the pupal decision as non-diapause development when adult eclosion occurred within 25 days from pupation. Cases where the plastic decision of individuals could not be assessed were excluded from the data, for instance, individuals that died during larval development (79 individuals in total (13.1%)) as well as individuals that died within the first 25 days of pupal development (five individuals in total (2%)).

To explore if *P. aegeria’s* two separate expansions into Sweden have evolved divergence in critical photoperiods based on a similar sex-linked effect, we directly compared results of the present study with the data from Pruisscher *et al.* [42]. As in the present study, Pruisscher *et al.* [42] assayed the diapause proportions of the larval decision of F1 offspring in reciprocal F1 population hybrids. Their results showed that the hybrid females with a father from the south (Skåne) had a markedly lower diapause proportion than hybrid females with a northern father (Sundsvall), while the males did not differ between reciprocal crosses. This indicated that genetic variation on the sex chromosomes influences the difference in the critical photoperiods of the Sundsvall and Skåne populations, which was also supported by whole-genome sequencing of these crosses. By directly comparing the results from the present study to this earlier study, it is possible to test if a similar sex-linked effect can explain at least parts of the difference in the critical photoperiods between the Stockholm and Gotland populations. However, while the present study measured the hybrids’ diapause response in considerable detail across the entire range of photoperiods between the critical photoperiods of the original populations, Pruisscher *et al.* [42] measured this response at one single photoperiod where the diapausing response of the two original populations was at either 0% or 100% diapause. For a direct comparison of the original data from the two studies, all data from the five photoperiods used in the present study were pooled within cross, resulting in one treatment per cross.

### (a) Statistical analysis

All analyses were carried out using R v. 4.1.2 for Windows and a *p*-value of 0.05 was applied throughout. The larval and pupal developmental decisions were scored using the criteria described above as either non-diapause (0) or diapause (1), adhering to binomial distributions. The link was set as ‘logit’. The photoperiodic treatment was analysed as a continuous variable to reflect its gradual change in nature. Confidence intervals for mean proportions in the figures were calculated as Wald CI (CI = *p *− *z* × s.e., *p* + *z* × s.e.).

In the analysis of the present comparison of Stockholm and Gotland, we first tested if the diapause response of the hybrids (independent of reciprocal cross) deviated from the response of the two founding populations by pooling the data from both reciprocal hybrid crosses. A generalized linear mixed-effect model with a logit link was fitted to the data to test if the response of diapause to variation in photoperiod was dependent on the main effects of cross (GG, SS and (GS + SG)), treatment (photoperiod) and sex (female, male). ‘Family’ was added as a random factor because there were up to three siblings in each treatment. During exploratory model analysis, models with interaction terms did not converge. Given our specific interest in determining whether the crosses exhibit variation in diapause response, we chose to construct a model containing solely the main factors and the random effect (electronic supplementary material, table S3). As the effect of the cross was significant, we performed a Tukey test using the emmeans package [73] to investigate which crosses differed from each other. Additionally, when the cross effect was significant, we estimated the variance explained by the cross by calculating a semi-partial *R*^2^ using the part R2 package [74], with 95% CI computed using 100 bootstrap interactions.

To answer our main question of whether sex-linked genes could explain some part of the difference in critical photoperiod between the Gotland and Stockholm populations, we tested if there was a difference in diapause response between the females from the two reciprocal hybrid crosses while we expect no such difference between the males from the reciprocal crosses (i.e. a significant sex × reciprocal cross interaction). We used a generalized linear mixed-effects model with a logit link and ‘family’ added as a random factor. As explanatory variables, we added the main effects of cross (GS and SG), treatment (photoperiod) and sex (female and male). Once more, during exploratory model analysis, the full model and models incorporating several interactions did not converge. Therefore, as the effect of the cross × sex interaction is the direct test of the hypothesis, it was the only interaction included in the final model (electronic supplementary material, table S3).

Finally, to directly test if the present study showed a pattern of sex-dependent inheritance that differed from the earlier study of the two, more distantly related, populations [42], we compared the data from both experiments. For this, we used the original data on F1 reciprocal hybrids from both studies and categorized each individual as having a father from the northern population of each cross (i.e. Sundsvall and Stockholm) or from the south (i.e. Skåne and Gotland). To investigate if the two studies showed similar patterns, we were interested in whether the sexes had similar diapause responses depending on their father’s origin in the two studies (a three-way interaction). Therefore, we used a generalized linear model (GLM) with a logit link and as explanatory variables, we added the factors: father’s origin (father from north or south), sex (male/female) and experiment (present/[42]). Model selection was performed by initially including all interactions and subsequently removing insignificant interactions to find the model with the lowest AIC value. However, in the final model, we included the interaction of father’s origin × sex × experiment, as it is the direct test of the hypothesis (electronic supplementary material, table S3).

In the experimental setup, the photoperiods were constant, which differs from the directional change in conditions that larvae experience in their natural environment. Therefore, during larval development, some individuals may have sensed the photoperiod as short enough for prolonged larval development time yet found the same photoperiod long enough to develop directly in the pupal stage (something that would almost never happen in the wild). To investigate the relationship between these decisions in our experiment, we calculated the correlation between larval and pupal decisions as the phi coefficient using the vcd package [71].

Since the individuals were scored as non-diapausing if their larval development time was below 40 days and some larvae occasionally get close to developing for 40 days, we performed a sensitivity analysis. This was done by repeating all the above-mentioned analyses but with individuals regarded as non-diapausing if their larval development time was below 36 days or 44 days ( ± 10%).

## 3. Results

**Figure 3.**
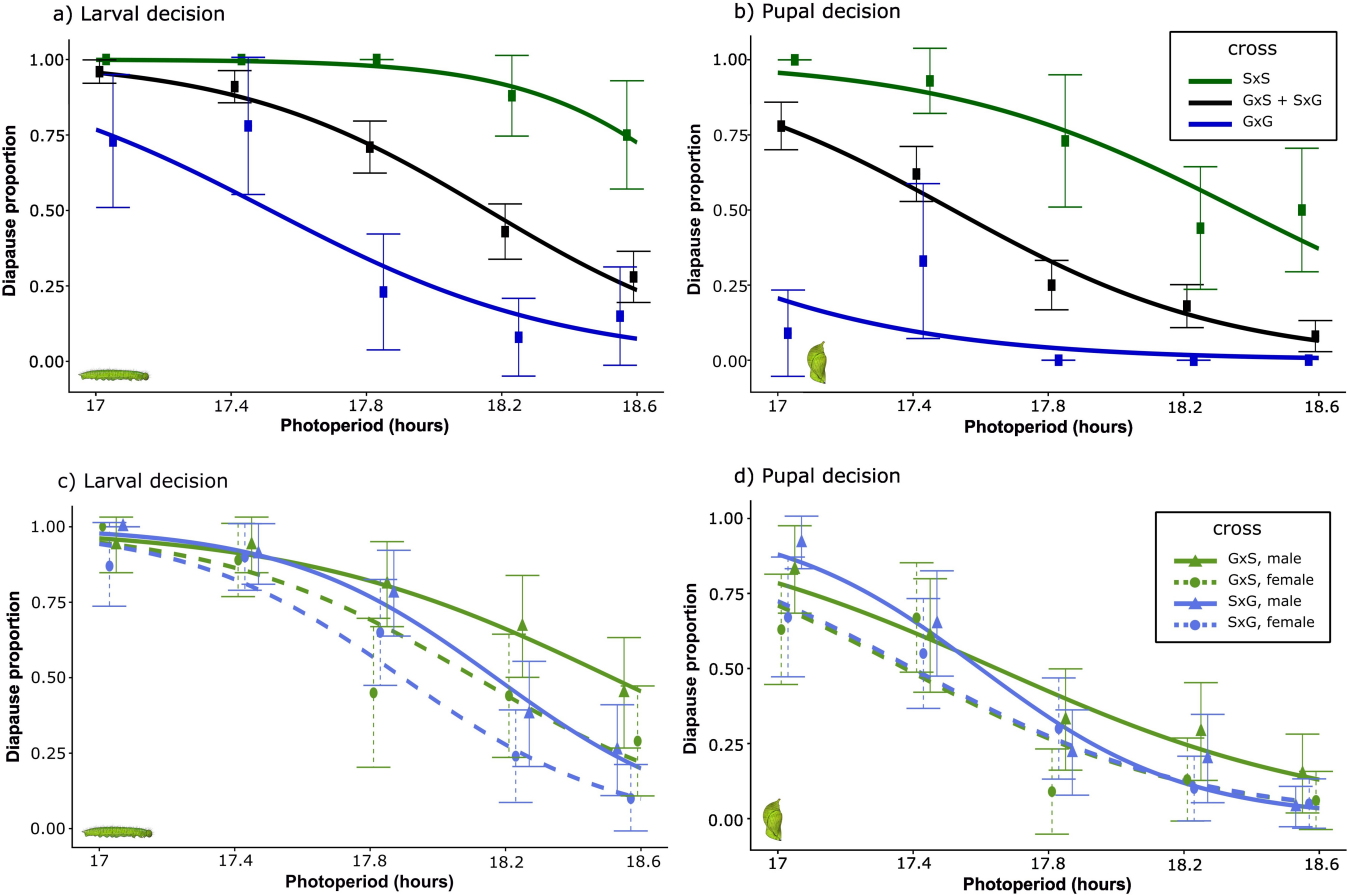
Proportion of diapausing individuals at the photoperiods 17, 17.4, 18.2, 18.2 and 18.6 h (left to right). All cabinets had a constant temperature of 18°C. Bars show 95% CI of actual proportions and functions show model-predicted responses. (a) Larval diapause response of the two original populations (G × G, Gotland, males and females, dark blue; S × S, Stockholm, males and females, dark green) and the reciprocal hybrid crosses pooled (G × S + S × G, males and females, black solid line). Means are depicted as rectangles based on 9–16 individuals for S × S and G × G and 75–80 individuals for G × S + S × G. (b) Same results but for the pupal decision. Means are based on 9–16 individuals for S × S and G × G and 75–80 individuals for G × S + S × G. (c) Larval diapause response for the two reciprocal crosses (G × S, father from Stockholm, light green; S × G, father from Gotland, light blue). Males have solid lines with means depicted as triangles, while females have stippled lines with means depicted as dots. Means are based on 11–19 individuals for G × S females, 18–21 individuals for G × S males, 15–21 individuals for S × G females and 21–26 individuals for S × G males. (d) Same results but for pupal decision. Means are based on 11–19 individuals for G × S females, 18–21 individuals for G × S males, 15–21 individuals for S × G females and 20–26 individuals for S × G males.

When the two reciprocal hybrid crosses were pooled, the diapause response of the F1 offspring was intermediate to the two natural populations across all five photoperiods, both for the larval decision to prolong larval development time and for the pupal decision to enter pupal diapause (figure 3a,b). The effect of F1 cross on diapause response was highly significant for both the larval and pupal decisions (cross; larval decision: χ^2^(2) = 36.24, *p* < 0.001, pupal decision: χ^2^(2) = 40.01, *p* < 0.001) and cross uniquely explained approximately 20% of the total variation in diapause decisions (larval decision: semi-partial *R*^2^ = 0.19, 95% CI: 0.03−0.39; pupal decision: semi-partial *R*^2^ = 0.22, 95% CI: 0.07−0.38). Additionally, performing a Tukey test revealed that the difference in diapause response between the crosses (GG, SS and (GS+SG)) was significant in all pairwise comparisons for both decisions (larval decision; GG-SS: *p* < 0.001, SS−(GS+SG): *p* < 0.001, GG−(GS+SG): *p* < 0.001, pupal decision; GG-SS: *p* < 0.001, SS−(GS+SG): *p* < 0.001, GG−(GS+SG): *p* < 0.001). This suggests that variation in both the larval and pupal decisions has a strong genetic basis. In addition, for both decisions there was a significant effect of sex (larval decision: χ^2^(1) = 17.41, *p* < 0.001, pupal decision: χ^2^(1) = 16.46, *p* < 0.001) and photoperiod treatment (larval decision: χ^2^(1) = 92.64, *p* < 0.001, pupal decision: χ^2^(1) = 88.79, *p* < 0.001). The difference in sex indicates that females generally enter diapause at a shorter photoperiod than males, which has previously been observed in this and other butterflies [75]. To investigate the hypothesis of a potential Z-linked effect on the difference in critical photoperiods between the Stockholm and Gotland populations, we next analysed the diapause responses of the two reciprocal hybrid crosses. Also, in this analysis, we tested the larval and pupal decisions separately (figure 3c,d). The cross : sex interaction was non-significant for both decisions (larval decision: χ^2^(1) = 0.30, *p* = 0.5810, pupal decision: χ^2^(1) = 0.44, *p* = 0.5052), indicating that genetic variation at the Z-chromosome did not contribute significantly to the difference in the diapause response between the Stockholm and Gotland populations. In both the larval and pupal decisions, the photoperiod treatment (larval decision: χ^2^(1) = 77.86, *p* < 0.001, pupal decision: χ^2^(1) = 77.19, *p* < 0.001) and sex (larval decision: χ^2^(1) = 12.73, *p* < 0.001, pupal decision: χ^2^(1) = 6.73, *p* = 0.0095) had significant effects on the diapause response, whereas there was no significant effect of cross in any of the decisions (larval decision: χ^2^(1) = 2.30, *p* = 0.1298, pupal decision: χ^2^(1) = 0.06, *p* = 0.8050). The difference between sexes indicates that, similarly to the original populations, the reciprocal hybrid females enter diapause at a shorter photoperiod than the males.

**Figure 4.**
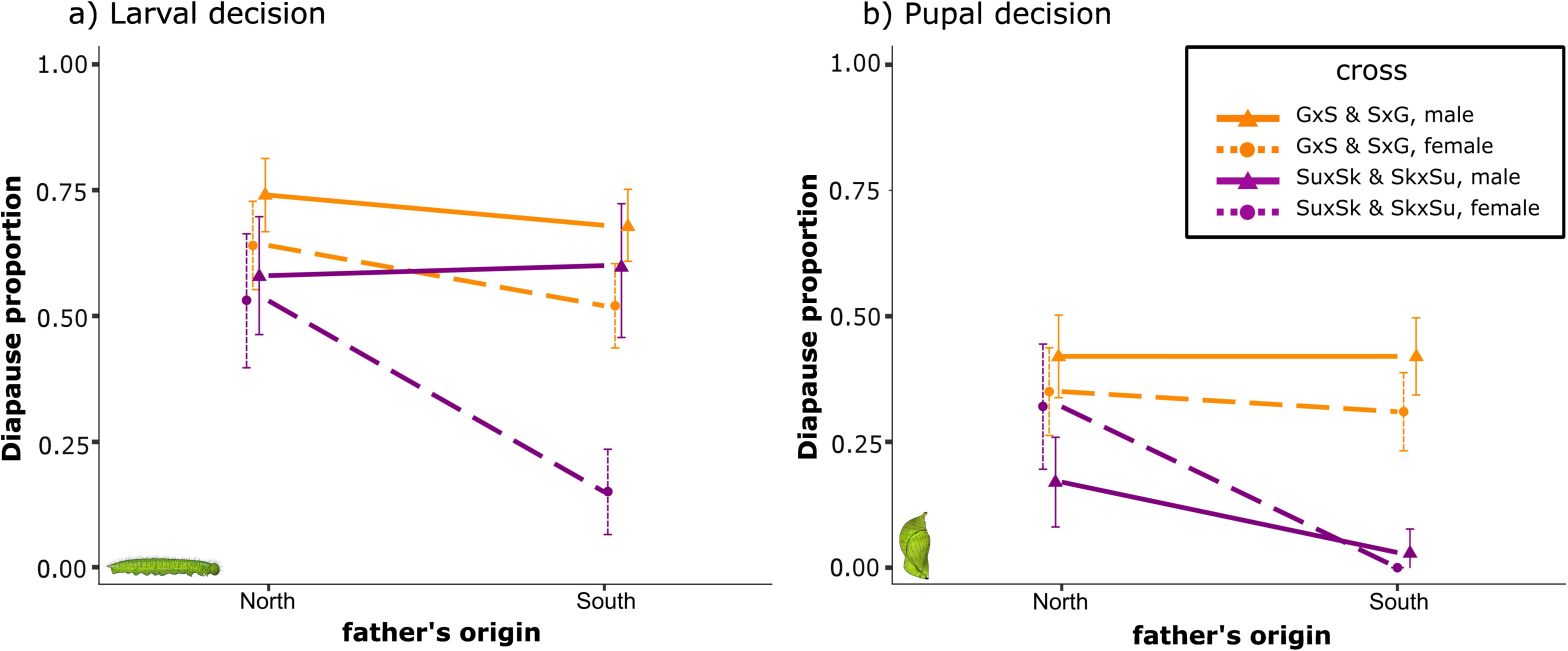
Proportion of diapausing individuals in reciprocal hybrid crosses from the present (orange) and the study by Pruisscher *et al.* [42] (purple). Within the studies, individuals are classified by father’s origin (Stockholm = north, Gotland = south; Sundsvall = north, Skåne = south) and by sex (females, stippled lines and dots; males, solid lines and triangles). Bars show 95% CI of actual proportions. (a) Larval decision. Means are based on 81 individuals for G × S females, 98 for G × S males, 96 for S × G females, 115 for S × G males, 38 for Sk × Su females, 48 for Sk × Su males, 48 for Su × Sk females and 37 for Su × Sk males. (b) Pupal decision. Means are based on 81 individuals for G × S females, 98 for G × S males, 96 for S × G females, 112 for S × G males, 38 for Sk × Su females, 48 for Sk × Su males, 47 for Su × Sk females and 36 for Su x Sk males.

Finally, we compared the findings from our reciprocal hybrid crosses (GS and SG) with the reciprocal hybrid crosses of the Sundsvall and Skåne populations investigated by Pruisscher *et al.* [42]. Since that study only measured the diapause response of the hybrids at one photoperiod, the five photoperiods from the present study were pooled for comparison. If the effect of genomic regions at the Z-chromosome on the diapause response identified by Pruisscher *et al.* [42] would also be involved in explaining the difference between the two populations we studied here, then we expect no significant three-way interaction between the origin of the father (north or south), sex and experiment. However, for the larval decision, we did find a significant three-way interaction (χ^2^(4) = 10.71, *p* = 0.0300), indicating that the results in the two experiments did differ (figure 4a). Hence, although parts of the difference in the critical photoperiod between the Sundsvall and Skåne populations can be explained by a Z-linked effect in their larval decision, the same does not seem to be the case for the difference in critical photoperiods between the Stockholm and Gotland populations. When testing the pupal decision, no significant three-way interaction was found (χ^2^(3) = 3.00, *p* = 0.3917), indicating that for the pupal decision, the reciprocal hybrids reacted similarly in the two studies, showing no Z-linked effect on diapause response (figure 4b). Instead, a significant two-way interaction was found between the origin of the father and the experiment (χ^2^(1) = 21.40, *p* < 0.001), suggesting that the diapause responses of individuals with a father from the south or the north did differ between the experiments but that this difference did not depend on the sex of the tested individual. Having a father from Skåne compared with Sundsvall (south and north in Pruisscher *et al.* [42]) affected the diapause response more than having a father from Gotland compared with Stockholm (south and north in the present study).

Development under a constant photoperiod may lead individuals to prolong larval development time but still develop directly as pupae [65]. In the present study, a strong positive correlation was observed between the two decisions (*r* = 0.58). Specifically, 179 individuals were scored as having both a short larval development time and developing directly as pupae, while 201 individuals were scored as having a long larval development time and entering diapause as pupae. Additionally, 136 individuals were scored as having a long larval development time but developing directly as pupae. No individuals exhibited the opposite pattern of having a short larval development time and entering diapause as pupae (electronic supplementary material, table S5). To confirm that the results for the larval decision were robust, a sensitivity analysis was performed. In the present study, individuals were regarded as non-diapausing if their larval development time was below 40 days. In the sensitivity analysis, the cutoff point was set to be either below 36 days or 44 days. This caused no difference in the results in any of the above-mentioned analyses (electronic supplementary material, table S4).

## 4. Discussion

A direct comparison of population crosses both within and between colonization events of *P. aegeria* (Gotland × Stockholm, Skåne × Sundsvall) suggests that similar adaptive phenotypic variation in photoperiodic plasticity has evolved repeatedly during separate colonizations, and that these differences are partly based on different genetic architectures. While the earlier cross between Skåne and Sundsvall shows a strong Z-linked effect on diapause induction, hybrid crosses between the populations from Gotland and Stockholm show no such effect.

The present and previous studies clearly show that local phenotypic variation in photoperiodic plasticity for diapause induction is to a large degree due to genetic variation across populations of *P. aegeria* [29,35,42,71]. This is true for both the photoperiodic plasticity of larval and pupal development, which together constitute the decision to induce winter diapause in the pupal stage in *P. aegeria*. Under common-garden conditions (photoperiods and temperature), the reaction norms of the photoperiodic response for the Stockholm and Gotland populations significantly differ (figure 3a,b). In line with the adaptive prediction, the critical photoperiod is highest for the univoltine population at the highest latitude (Stockholm), meaning that individuals of this population enter diapause development earlier in the summer compared to the Gotland population [38]. In both larval and pupal plasticity, the F1 hybrid crosses have an intermediate reaction norm (figure 3a,b) which is in accordance with general expectations of polygenic inheritance [25,37,76].

The results demonstrate that only variation in the reaction norm for larval development shows Z-linked inheritance in one of the population pairs, while there is no evidence for a similar effect on the plasticity of pupal diapause (figure 4). This suggests that the two decisions rely on partly different genetic mechanisms. In line with this, the larval and pupal reaction norms have previously been shown to evolve at different rates. The last 30+ years of climate change have led to the evolution of the larval reaction norm in line with adaptive predictions in one population while having no significant effect on the pupal reaction norm in two separate Swedish populations [71].

Previous whole-genome sequencing has demonstrated that variation in the critical photoperiod across populations of *P. aegeria* in Sweden is associated with variation at genomic regions containing genes of the circadian clock [42,62], which is also a pattern found in many other insects [41,47,49,77]. In particular, genomic regions containing the genes period and timeless have been associated with the difference in critical photoperiod between the Skåne and Sundsvall populations [42]. With special relevance for the present study, the period gene is located on the Z-chromosome in *P. aegeria* and variation within this gene may partly explain the sex-linked effect demonstrated in the study of Pruisscher *et al.* [42]. In contrast, the present study suggests that any influence of genetic variation at the Z chromosome is of less importance for explaining the difference in photoperiodic plasticity between the two more closely related populations from Stockholm and Gotland. The other circadian clock gene, timeless, is instead situated on an autosomal chromosome [42] and interestingly, the Gotland population is fixed for a relatively large deletion in the timeless gene not found in any of the other populations [62]. It is interesting to speculate that this deletion may influence the photoperiodic response of the Gotland population. If so, it is possible that genetic variation for different clock genes may contribute to similar phenotypic variation across *P. aegeria* populations, with natural selection favouring distinct combinations of genetic variants in different locations.

It is often expected that repeated phenotypic evolution is caused by variation within the same genes, especially across populations of the same species [6]. This is because closely related populations are likely to share the same pool of standing genetic variation on which natural selection acts. However, as time since divergence increases, gene reuse is likely to decrease [7]. In the present case, the absolute divergence times are not known, but it is clear that the divergence of the most southern population (Skåne) is older than between any other of the Swedish populations. The present results suggest that the photoperiodic response for diapause induction has evolved through selection on partly different genetic variation across populations of *P. aegeria* in Sweden. It is conceivable that the photoperiodic response for diapause induction is influenced by a large number of genes of small effect that were segregating in the ancestral populations, making it more likely that the same phenotypic variation may evolve by different genetic variation [5]. Alternatively, the difference in demographic history across these populations may have resulted in a difference in the standing genetic variation at the time of the two expansions into Scandinavia. For instance, it is possible that the very recent invasion into southern Sweden may have brought genetic variation at the Z chromosome that evolved further south in Europe, after the initial expansion into Scandinavia. The first and much older expansion would then have adapted through selection on standing genetic variation at other chromosomes or mutations that arose during the northward expansion. It should be noted that there is a quantitative difference in how the timing of diapause induction varies between the two population pairs (the difference in the critical photoperiod between Stockholm and Gotland ≈ 1.4 h, and between Sundsvall and Skåne ≈ 3 h). It is possible that the divergence in the photoperiodic timing of diapause induction of the magnitude seen between the two extreme populations in Sweden requires additional genetic changes that may be associated with the evolution of Z-linked genes. Future studies of the phenotypic effects of alternative alleles at the autosomes using genetic backcrosses or gene editing in this quite tractable system could offer valuable insight into the genotype–phenotype map of photoperiodism in *P. aegeria* and for insects in general.

Although it is frequently observed that closely related populations and species show parallel genetic backgrounds to parallel adaptive phenotypes, it is clear that alternative genetic variation may also underpin similar phenotypes [6,7]. The last option seems to be the case for photoperiodic plasticity for diapause induction in *P. aegeria*. We built on previous genomic insights showing that candidate regions are situated on the Z chromosome, to explore how genetic variation at this chromosome affects variation in photoperiodic plasticity for diapause induction. As the differences in the diapause response were Z-linked in one population pair but not the other, we conclude that the evolution of similar phenotypic variation has evolved by partly different genetic mechanisms. This shows that the genotype–phenotype map may not be constant across a species range, even for traits that appear to evolve relatively rapidly to form local adaptations across populations.

## Data Availability

The data supporting the findings of this study are openly available in the Dryad repository [78]. Supplementary material is available online [79].
